# Unlocking biological complexity: the role of machine learning in integrative multi-omics

**DOI:** 10.20935/acadbiol7428

**Published:** 2024-11-27

**Authors:** Ravindra Kumar, Rajrani Ruhel, Andre J. van Wijnen

**Affiliations:** 1 Department of Psychiatry, Washington University in Saint Louis School of Medicine, Saint Louis, MO 63110, United States.; 2 Department of Developmental Biology, Washington University in Saint Louis School of Medicine, Saint Louis, MO 63110, United States.; 3 Department of Biochemistry, University of Vermont, Burlington, VT 05405, United States.

**Keywords:** machine learning, multi-omics, data integration

## Abstract

The increasing complexity of biological systems demands advanced analytical approaches to decode the underlying mechanisms of health and disease. Integrative multi-omics approaches use multi-layered datasets such as genomic, transcriptomic, proteomic, and metabolomic data to understand biological processes much more comprehensively compared to the single-omics analysis and to provide a comprehensive view of cellular and molecular processes. However, these integrative approaches have their own computational and analytical challenges due to the large volume and nature of multi-omics data. Machine learning has emerged as a powerful tool to help and resolve these challenges. It offers sophisticated algorithms that can identify and discover hidden patterns and provide insights into complex biological networks. By integrating machine learning in multi-omics, we can enhance our understanding of drug discovery, disease, pathway, and network analysis. Machine learning and ensemble methods allow researchers to model nonlinear relationships and manage high-dimensional data, improving the precision of predictions. This approach paves the way for personalized medicine by identifying unique molecular signatures for individual patients, which can provide valuable insights into treatment planning and support more effective treatment. As machine learning continues to evolve, its role in multi-omics analysis will be pivotal in advancing our ability to interpret biological complexity and translate findings into clinical applications.

With the advent of multi-omics technologies, the field of biomedical and human health research has witnessed a major shift. These multi-omics technologies allow the integration of diverse data encompassing genomics, epigenomics, transcriptomics, proteomics, glycoproteomics, glycomics, and metabolomics, which collectively allow scientists to capture a comprehensive view of the molecular underpinnings of biological systems [1–3]. This presents unprecedented opportunities to unravel the complexity of diseases [1, 4], identify and discover novel biomarkers [1, 4], and create new and meaningful biological knowledge [2]. However, the sheer volume and heterogeneity of multi-omics data pose significant analytical challenges [2]. This is where machine learning (ML) emerges as a powerful artificial intelligence–related tool. ML applies algorithms that learn from data to make predictions or uncover patterns without being explicitly programmed [5]. ML algorithms enable the processing and analysis of vast and complex multi-omics datasets by identification of hidden relationships and provide insights that are often beyond human capability. The synergy between ML and multi-omics holds immense promise for advancing our understanding of biology and improving clinical outcomes [6].

One of the primary goals of ML in multi-omics is the integration and interpretation of data from different omics layers [7]. Traditional statistical methods often struggle with the high dimensionality and noise that is inherent in multi-omics datasets. ML algorithms, particularly deep learning models, excel in handling such high complexity. They can integrate data from various sources to build holistic models of biological systems, thereby providing a more comprehensive understanding of cellular processes and disease mechanisms.

Another significant application of ML in multi-omics is biomarker discovery [8]. Identifying biomarkers for disease diagnosis, prognosis, and treatment response is crucial for precision medicine. ML algorithms can sift through multi-omics data to pinpoint molecular signatures associated with specific phenotypes or clinical outcomes. For instance, ML models have been used to identify gene expression patterns linked to cancer subtypes, protein markers indicative of cardiovascular disease [9], neurodegenerative diseases [10–12], and metabolite profiles associated with metabolic disorders.

ML has also revolutionized the field of drug discovery and development [11]. By consolidating multi-omics data with chemical and pharmacological information, ML models can predict drug–target interactions [13–15], identify potential drug repurposing opportunities [16], and forecast adverse drug reactions. This capability of ML accelerates the drug development pipeline and enhances the likelihood of success in clinical trials.

Despite these advances, the application of ML in multi-omics data analysis is not without challenges. The quality and standardization of omics data remain critical issues. Variability in data acquisition, processing, and annotation can lead to inconsistencies and biases that affect ML model performance. Additionally, the interpretability of ML models, especially deep learning algorithms, is a significant concern. Although these models can uncover complex patterns, understanding how they make predictions and ensuring their decisions are biologically plausible are crucial for their acceptance in the biomedical community.

Ethical considerations also come into play when applying ML to multi-omics data. The use of patient data necessitates stringent privacy and security measures. Furthermore, ensuring that ML models do not perpetuate existing biases in healthcare is essential for promoting equity and fairness in medical research and treatment.

In conclusion, the integration of ML with multi-omics data analysis represents a transformative approach in biomedical research. The ability of ML to handle large complex datasets with missing values and to uncover hidden patterns renders ML an invaluable tool for understanding the intricacies of biological systems and advancing precision medicine. As we continue to refine ML algorithms and address the associated challenges, the synergy between ML and multi-omics will undoubtedly drive significant breakthroughs in science and medicine. The future of healthcare lies in this powerful convergence, promising more accurate diagnoses, personalized treatments, and ultimately, better patient outcomes.

## Data Availability

Data supporting these findings are available within the article, at https://doi.org/10.20935/AcadBiol7428, or upon request.
